# Effect of Cold Atmospheric Pressure Plasma Coupled with Resin-Containing and Xylitol-Containing Fluoride Varnishes on Enamel Erosion

**DOI:** 10.1155/2021/3298515

**Published:** 2021-07-27

**Authors:** Zahra Khoubrouypak, Mahdi Abbasi, Elham Ahmadi, Niyousha Rafeie, Marjan Behroozibakhsh

**Affiliations:** ^1^Department of Operative Dentistry, School of Dentistry, Guilan University of Medical Sciences, Rasht, Guilan, Iran; ^2^Department of Operative Dentistry, School of Dentistry, Tehran University of Medical Sciences, Tehran, Iran; ^3^Dental Research Center, Dentistry Research Institute, School of Dentistry, Tehran University of Medical Sciences, Tehran, Iran; ^4^Department of Dental Biomaterials, School of Dentistry, Tehran University of Medical Sciences, Tehran, Iran

## Abstract

**Purpose:**

Considering the suggested advantages of cold atmospheric plasma (CAP) in increasing the fluoride uptake by the enamel, this study aimed to assess enamel erosion following the application of helium CAP and two types of fluoride varnishes.

**Methods:**

The microhardness of 70 bovine enamel specimens was measured using a Vickers hardness tester. The specimens were randomly divided into 7 groups (*n* = 10): control, CAP (P), resin-containing fluoride varnish (RF), CAP + resin-containing fluoride varnish (PRF), fluoride varnish (F), CAP + fluoride varnish (PF), and erosion (E). The specimens in the control and erosion groups did not receive CAP or fluoride varnish. All specimens underwent erosive challenge 4 times/day using hydrochloric acid and artificial saliva except for the control specimens that remained in distilled water during the course of the study. After 5 days of erosive challenge, microhardness was measured again, and the percentage of microhardness change was calculated. Surface roughness of two specimens in each group was assessed by atomic force microscopy (AFM). Data were analyzed using one-way ANOVA followed by Tamhane's post-hoc test.

**Results:**

The percentage of microhardness change in all groups was significantly higher than that of the control group. All groups showed significantly lower percentage of microhardness change compared with the E group except for the P group; no significant difference was noted in microhardness change of P and E groups. Other experimental groups had no significant difference with each other. Surface roughness was the highest in PRF and the lowest in the F group.

**Conclusion:**

CAP application had no significant effect on increasing the enamel resistance to erosion. However, enamel resistance to erosion increased significantly after fluoride varnish application alone or fluoride varnish application combined with CAP. No significant difference was noted between the two types of varnishes in this regard. CAP increased the surface roughness while fluoride varnish application alone decreased the roughness.

## 1. Introduction

Dental erosion refers to the progressive and irreversible loss of the tooth structure by chemical processes without the involvement of bacteria [1]. This process can be caused by exposure to intrinsic or extrinsic acids. Acidic foods and beverages are the sources of extrinsic acids while esophageal reflux, chronic inflammation of the gastrointestinal system, bulimia, and anorexia are considered as the sources of intrinsic acids because they lead to frequent vomiting [2]. Gastritis, poor oral hygiene, and the presence of orthodontic brackets are among other predisposing factors to dental erosion [3]. Dental erosion has a multifactorial etiology, encompassing a wide range of chemical, biological, and behavioral factors. Its clinical manifestations include the loss of tooth surface anatomy, increased incisal translucency, and incisal enamel chipping [4, 5]. The prevalence of dental erosion ranges from 27% to 83% [6] and often increases with age due to prolonged exposure to erosive factors [7, 8].

In the past two decades, fluoride varnishes have become highly popular due to advantages such as widespread acceptance, easy application, and high-level safety [9]. Application of fluoride to enhance the resistance of tooth structure to acid attacks has long been acknowledged. Evidence shows that fluoride can prevent, stop, and even reverse the process of demineralization. A previous study showed that sodium fluoride (NaF) significantly decreased dental erosion caused by exposure to citric acid (pH of 2.6) [10]. The protective effect of fluoride varnishes is mainly attributed to the adhesion of varnish to the tooth surface, creating a mechanical barrier and increasing the contact time of fluoride with the tooth surface [11]. Fluoride varnishes may contain resin-modified glass ionomer, xylitol, calcium, and phosphate; such varnishes often show more favorable results than the conventional fluoride varnishes [12].

FluoroDose is a fluoride varnish containing 5% NaF and xylitol, while Enamelast is another fluoride varnish containing a resin component in addition to xylitol as claimed by the manufacturer.

Wegehaupt et al. [13] reported that the application of resin-based sealants significantly decreased enamel demineralization following exposure to hydrochloric and citric acids. It is believed that resin infiltrate can penetrate into the porous enamel structure and prevent further acid diffusion [14]. Enamelast varnish contains a patented component, which is believed to improve the contact time between the tooth surface and the varnish, consequently improving the formation of CaF2 on the enamel [15]. We also chose FluoroDose as our study material because it contains the same amount of fluoride and xylitol present in Enamelast varnish; however, FluoroDose lacks the resin component in formulation as opposed to Enamelast. Besides, FluoroDose varnish is less sticky and its color is similar to that of natural teeth, which makes it a practical choice for clinical application [16].

Plasma is a highly active form of material that contains radicals, high-energy ions, free electrons, strong electric fields, and charged particles [17]. In recent years, cold atmospheric plasma (CAP) has been used for root canal sterilization, enhancement of bond strength, and tooth bleaching [18–20]. Kim et al. [21] showed that helium CAP prior to varnish application enhanced the uptake of fluoride ions by the enamel surface. Moreover, evidence shows that the effects of CAP treatment in combination with fluoride last for a longer period of time compared with the effects of fluoride alone. It is hypothesized that CAP application may improve the efficacy of fluoride varnishes by two main mechanisms; first mechanism is that high level of plasma energy might separate the fluoride from fluoride compounds [22]. Thus, it is possible that applying CAP in combination with fluoride varnishes improves fluoride release from varnishes and improves their efficacy in formation of CaF2, which subsequently increases the enamel resistance to erosion. The second mechanism is that CAP application significantly modifies the hydrophilic properties of dental surfaces and improves the surface wettability by removing some of the proteins from the enamel surface [23]. We assumed that fluoride uptake and retention after CAP application might increase due to improved wettability of enamel surfaces.

At present, considering the increased prevalence of dental erosion, several strategies are employed to prevent or control dental erosion, all aiming to remineralize the tooth structure and prevent the progression of erosive lesions.

Various techniques are used to assess enamel erosion. Measuring the surface microhardness by the microindentation technique is a popular method for assessing dental erosion due to its easy use and low cost [24].

Considering the novelty of the combined use of CAP and fluoride varnishes, the presence of evidence supporting increased fluoride retention following the application of helium CAP, and the resin component in the formulation of Enamelast varnish, which has been claimed to increase the contact time of the varnish with tooth, this study aimed to assess enamel erosion following the application of CAP and two types of resin-containing and xylitol-containing fluoride varnishes. The first null hypothesis was that CAP application would have no significant effect on enamel resistance to erosion. The second null hypothesis was that the combined application of CAP and Enamelast or FluoroDose would have no significant effect on enamel resistance to erosion.

## 2. Materials and Methods

In this experimental study (ethical approval code: IR.TUMS.DENTISTRY.REC.1398.142), the minimum sample size was calculated to be 10 specimens in each experimental group according to a similar study by Passos et al. [25] using one-way ANOVA feature of PASS 11 software (NCSS, LLC., Kaysville, Utah, USA), considering alpha = 0.05, beta = 0.2, mean standard deviation of microhardness equal to 6, and the effect size of 0.44.

A total of 70 bovine incisors without caries or discoloration were collected. The soft tissue residues were removed using a gauze, and calculus was removed using a hand scaler. The teeth were then disinfected by immersion in 0.5% chloramine *T* solution for 1 week [26]. Then, the teeth were decoronated at 3 mm below the cementoenamel junction by a diamond metal disc (North Bel Co., Paderno Dugnano, Italy). The coronal sections were mounted in cylindrical molds containing transparent auto-polymerizing acrylic resin (Acropars Co., Tehran, Iran). The buccal surface of the teeth remained exposed out of the acrylic resin and polished with 400, 600, and 1200-grit abrasive papers (Smirdex Co., Xánthi, Greece) using a polishing machine (Dorsa Co., Tehran, Iran) [27]. After polishing, the specimens were cleaned in an ultrasonic bath (COLLIN20, Woson Medical Instrument Co., Ningbo, China) for 10 min.

The baseline microhardness of the specimens was measured using a Vickers indenter (Bareiss Co., Stuttgart, Germany). For this purpose, 50 g load was applied for 5 s to create three indentations at 3 points, 100 µm apart from each other. The mean of the three values was calculated, and the specimens with a mean Vickers hardness number of 350 ± 43 were selected [28, 29].

The selected specimens were then randomly assigned to 7 groups (*n* = 10). In the control group, the specimens remained in deionized distilled water until the end of the experiment. In the erosion group (E), the specimens underwent erosive challenge without any intervention (neither fluoride varnish nor CAP application). The experimental groups were as follows: P: CAP, RF: resin-containing fluoride varnish (Enamelast varnish), PRF: CAP + Enamelast, F: fluoride varnish (FluoroDose varnish), and PF: CAP + FluoroDose.

**Figure 1:**
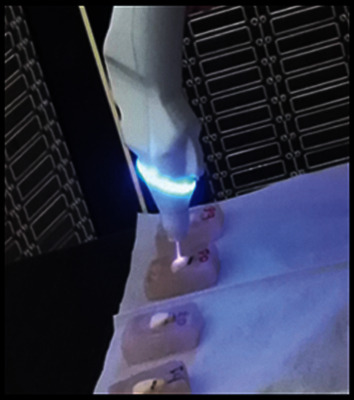
Application of CAP on the specimens.

Prior to the erosive challenge, the specimens in the CAP groups underwent treatment with a plasma jet (Medaion, Nik Fanavaran Plasma Co., Tehran, Iran) using helium gas at a flow rate of 2 L/min with a potential difference of 4 kV, and 20 kHz frequency such that the nozzle tip had a maximum distance of 10 mm from the tooth surface [21] (Figure 1). Next, the specimens were subjected to different fluoride varnishes according to their experimental groups.  Table 1 presents the composition of fluoride varnishes used in this study. Fluoride varnishes were applied in one thin coat by a microbrush according to the manufacturers' instructions. The specimens were then immersed in artificial saliva for 6 h, and eventually, fluoride varnishes were removed by a combination of acetone and water [30].

Next, the specimens underwent erosive challenge; enamel specimens were immersed in centrifuge test tubes containing 0.01 M hydrochloric acid with a pH of 2.3 for 2 min to undergo demineralization. The specimens were then rinsed with distilled water for 5 s and placed in centrifuge tubes containing artificial saliva (0.213 g/L CaCl2^*∗*^2H2O; 0.738 g/L KH2PO4; 1.114 g/L KCl; 0.381 g/L NaCl; 12 g/L Tris Buffer, pH adjusted to 7.0 with concentrated HCl solution) for 2 h. The composition of artificial saliva was similar to that in the study by Viana et al. [31]. The erosive challenge was repeated 4 times a day, for 5 consecutive days. Moreover, the specimens underwent abrasion with an electric toothbrush (Oral-B, Braun GmbH, Kronberg, Germany) and nonfluoridated toothpaste (Chicco Co., Lombardy, Italy) with water/toothpaste ratio of 3 : 1 by volume for 2 min. We designed a custom-made holder for the electric toothbrush. The toothbrush was fixed in the holder in such a way that the bristles were in contact with the surface of specimen. Moreover, the toothbrush had a pressure sensor, which would signal the operator once the applied pressure reached 2 N. The abrasion cycle was performed twice a day after the first and the last erosive challenge in each day. Finally, the specimens were stored in artificial saliva overnight (14 h). The specimens were kept at room temperature during this process, and the acidic solution and artificial saliva were refreshed daily [14, 30].

After the 5th day of the erosive challenge, the final microhardness of the specimens was measured as explained earlier. The percentage of microhardness change was calculated for each enamel specimen using the following equation:

(1) $$\begin{array}{c} \text{Surface microhardness change }=\frac{\text{SMH baseline}-\text{SMH final}}{\text{SMH baseline}}\times 100, \end{array}$$

where SMH baseline is the baseline microhardness prior to the erosive challenge and SMH final is the final microhardness after the erosive challenge.

To assess the surface roughness of the specimens, two enamel specimens were randomly selected from each group and underwent atomic force microscopy (AFM; Brisk, ARA Research Co., Tehran, Iran) in noncontact mode after surface treatments and 5 days of erosive challenge. It should be noted that AFM analysis was only performed to assess the surface roughness qualitatively.

Data were analyzed using SPSS version 25 (IBM Corp. Released 2017. IBM SPSS Statistics for Windows, Version 25.0, Armonk, NY: IBM Corp.). Considering the normal distribution of the data verified by the Kolmogorov–Smirnov test, one-way ANOVA was applied for general comparison of the groups followed by Tamhane's post-hoc test for pairwise comparisons at 0.05 level of significance.

## 3. Results

**Figure 2:**
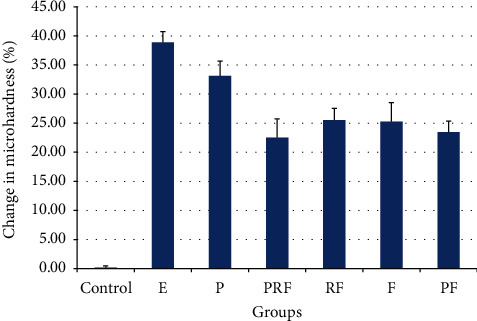
Mean and 95% confidence interval of the change in microhardness in different groups: E, Erosion, P CAP, PRF: CAP + Enamelast, RF: Enamelast, F FluoroDose, PF: CAP + FluoroDose.

Table 2 shows the descriptive evaluation of the groups. The minimum percentage of change in microhardness was noted in the control group (0.18 ± 0.91), while the maximum change was found in the E group (38.89 ± 5.88) followed by the P, RF, F, PF, and PRF groups, in decreasing order (Figure 2). According to one-way ANOVA, the difference in this respect was significant among the groups (*P* < 0.05). Thus, Tamhane's post-hoc test was applied for pairwise comparisons of the groups regarding microhardness. The percentage of change in microhardness of all groups was significantly higher than that of the control group (*P* < 0.001). All experimental groups showed significantly lower percentage of change in microhardness compared with the E group (*P* < 0.05), except for the P group; the percentage of change in microhardness of groups P and E was not significantly different with each other. Moreover, the other experimental groups had no significant difference with each other in this respect (*P* > 0.05).

**Figure 3:**
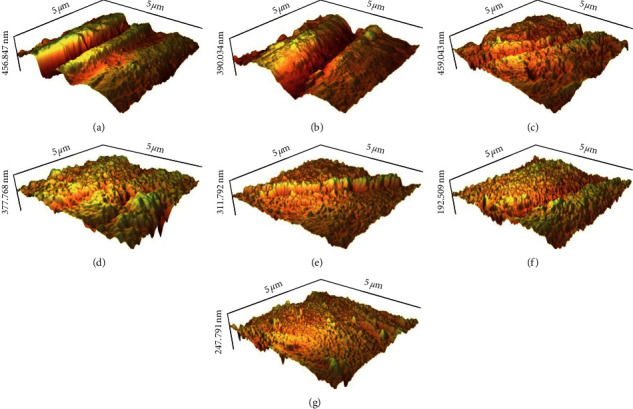
Atomic force microscopy image of enamel surface in groups after surface treatment and erosive challenge: (a) CAP + Enamelast, (b) CAP + FluoroDose varnish, (c) CAP application, (d) Erosion, (e) Control, (f) Enamelast varnish, and (g) FluoroDose varnish.

Evaluation of the enamel surface roughness by the AFM following the erosive challenge based on the Ra data revealed the maximum surface roughness in the PRF group followed by PF, P, E, Control, RF, and F in decreasing order (Figure 3).

## 4. Discussion

This study assessed the enamel erosion following the application of CAP and resin-containing and xylitol-containing fluoride varnishes. The results showed that the application of fluoride varnish with/without CAP significantly decreased the enamel erosion. No significant difference was noted between the Enamelast and FluoroDose fluoride varnishes in this respect. On the contrary, application of CAP alone did not improve enamel resistance to erosion. This result was in contrast to the findings of Kim et al. [21] because they showed that CAP enhanced the retention of fluoride.

Carvalho et al. [32] demonstrated that although fluoride varnishes released fluoride and played a role in initial formation of globular calcium fluoride, they could not induce remineralization and had no protective effect against erosion. They added that varnishes can only create a physical barrier against acid attacks. Their results were different from ours because in our study, fluoride varnishes had a protective effect on the enamel. The major difference between the study by Carvalho et al. [32] and this study is the different brands and composition of varnishes used. They used Duraphat and Duofluorid with 2.26% and 2.71% fluoride concentration, respectively, and two other experimental varnishes containing 5.63% fluoride plus 5% or 1% CaGP. Dissimilar erosive cycles and differences in the composition of the remineralization solution and fluoride varnishes used in the studies may explain the controversy in the results.

Regarding surface roughness, the AFM results in this study showed increased surface roughness in all CAP groups. However, Lehmann et al. [33] reported that the application of helium CAP had no significant effect on the alteration of the enamel surfaces. These differences might be due to the different methods used for evaluation of enamel surface topography.

Moreover, in our study, the groups treated with varnish alone showed lower surface roughness; these results supported the findings of Soares and De Carvalho Filho [34] who reported that fluoride protects the enamel but prevents an increase in Ra value after exposure to extrinsic acids. The Enamelast groups with/without CAP showed higher surface roughness than the FluoroDose groups, which may be due to the presence of citric acid in the composition of Enamelast.

We used bovine teeth instead of human teeth in this study because achieving an adequately large and smooth enamel surface for microhardness test and AFM was easier in bovine teeth due to their larger size. Although some morphological differences exist between the bovine and human teeth, such as a higher rate of porosities in bovine enamel compared with human teeth, a direct correlation has been observed between the microhardness and wear resistance of human and bovine enamel, which justifies the use of bovine teeth in this study [35]. Moreover, the enamel microhardness was measured using the microindentation method. Although other methods such as profilometry and microradiography are recommended for advanced assessment of enamel erosion, methods with lower technical sensitivity, such as microindentation, are recommended for assessment of primary erosion. Furthermore, we used artificial saliva for the remineralization process, which can effectively strengthen the softened enamel and is suitable for such in vitro studies [36]. It should be noted that polishing of the specimens in this study might have made them more susceptible to erosion compared with clinical conditions. However, polishing was necessary to standardize the specimens and provide a test surface with uniform composition [35].

Plasma jets use different gases such as helium, argon, and oxygen. Helium gas was used in this study with ionization energy of 24.5 eV. Helium has a lower ionization rate than argon and better thermal conductivity, preventing thermal instability [37]. Furthermore, surface treatment with plasma jet has been suggested to enhance recrystallization of minerals [26]. It can be used to produce nano-scale crystals with high quality and improve the dissolution speed [38, 39]. Lehmann et al. [33] showed that application of helium plasma on the enamel surface significantly decreased the carbon content, resulting in a relative increase in concentration of calcium, phosphorous, and oxygen. Šantak et al. [40] reported that the Ca/P ratio of the enamel increased following plasma treatment and reached 1.7, which was highly close to 1.67, which is the maximum reported remineralization volume and the highest rate of remineralization of human enamel. Owing to the aforementioned beneficial effects of helium plasma, we decided to evaluate its potential effect combined with fluoride varnish application on enamel microhardness after simulation of acid erosion.

We applied acetone and brushed the samples with a toothbrush and nonfluoridated toothpaste to better simulate a clinical setting in which the varnish on tooth surfaces is removed by mastication and daily brushing. The purpose behind the use of nonfluoridated toothpaste was to eliminate its synergistic effect with the fluoride varnishes. However, application of acetone may have an adverse effect on the resin component of Enamelast, preventing its optimal efficacy in increasing the retention of fluoride; although, it appears that the reinforcing effect of xylitol may compensate for it, as described by Çetin et al. [41].

The results of scanning electron microscopic/energy dispersive X-ray spectroscopic analyses revealed that less than 1% of fluoride remained on the surface after erosion or erosion/abrasion. Such a low percentage may not have any protective effect against the erosion or erosion/abrasion [34, 42]. Thus, it appears that application of helium CAP and fluoride varnishes does not significantly change the Ca/P ratio, and the percentage of residual fluoride on the enamel surface after erosion and abrasion is insignificant. It appears that the effect of xylitol was stronger than the combined protective effect of fluoride varnish and CAP in our study.

Xylitol forms a complex with calcium in the tooth structure and enhances the remineralization potential under in vitro erosive conditions [43]. In other words, xylitol serves as a source of calcium storage [41]. Both Enamelast and FluoroDose fluoride varnishes contain xylitol, which may explain their optimal efficacy because xylitol stabilizes the calcium during the demineralization cycles as explained by Souza et al. [44].

It should be noted that the current results were obtained after a 5-day pH cycling.

According to Wegehaupt et al. [13] a 6-min erosive cycle with hydrochloric acid at a pH of 3.0 simulates one day of intraoral environment. For instance, in patients with gastroesophageal reflux, the pH decreases to values < 5.5 for 4.3 min during 24 h [45]. In this study, hydrochloric acid with a pH of 2.3 was used, and the specimens were immersed in it for 40 min. Thus, a 5-day pH cycling may simulate 10 or more days of intraoral environment.

The protective role of the saliva in limiting the extension of erosive lesions [46] and the role of dental plaque, pellicle, and soft tissue in retention and storage of fluoride were not considered in this study due to its in vitro design, which was a limitation [24]. Moreover, the presence of pellicle on the specimens decreases the microindentation hardness. Not using an acquired pellicle in our study was also a limitation. Also, hydrochloric acid without pepsin was used to simulate the gastric acid in this study; however, it has been demonstrated that although pepsin has no adverse effect on enamel demineralization, it has a negative effect on the efficacy of fluoride [47]. Thus, generalization of the results obtained from this study to the clinical setting must be done with caution.

Future studies should assess the effect of fluoride varnishes in combination with CAP on enamel microhardness following exposure to exogenous acids, and hydrochloric acid plus pepsin. The amount of released calcium and phosphorous in exposure to acidic solutions following the application of fluoride varnish and CAP should also be quantified.

In addition to fluoride varnishes, other materials with remineralizing potentials such as biomimetic hydroxyapatite [3] and phosphopeptide amorphous calcium phosphate [48] have exhibited promising results. Further studies evaluating the remineralization potential of these materials combined with CAP are recommended using qualitative and quantitative analyses.

## 5. Conclusion

Within the limitations of this study, we concluded that regardless of the application of CAP, both types of fluoride varnishes used in this study improved the enamel resistance to erosion. CAP had no significant effect on enamel resistance to erosion.

## Figures and Tables

**Table 1:** Composition of fluoride varnishes used in this study.

Fluoride varnish	Composition	Manufacturer	Manufacturing country
Enamelast	Synthetic resin (<50), ethyl alcohol (<15), sodium fluoride (<5), methyl ester of hydrogenated rosin citric acid (<3), xylitol, 22,600 ppm F	Ultradent products	USA
FluoroDose	5% sodium fluoride varnish with xylitol, 22,600 ppm F	Centrix	USA

**Table 2:** Measures of central dispersion for change in microhardness of the groups.

Group	Mean ± SD	Maximum	Minimum
Control	0.18 ± 0.91^a^	2.55	−0.59
E	38.89 ± 5.88^c^	46.03	29.93
P	33.12 ± 8.02^c^	42.70	24.04
PRF	22.53 ± 10.19^b^	39.63	6.79
RF	25.53 ± 6.30^b^	36.08	11.40
F	25.24 ± 10.31^b^	37.50	8.90
PF	23.45 ± 5.98^b^	31.55	14.30

SD: Standard deviation, E: Erosion, P: CAP, PRF: CAP + Enamelast, RF: Enamelast, F: FluoroDose, PF: CAP + FluoroDose. Different lowercase letters indicate significant differences (*p* < 0.05). The same lowercase letters indicate lack of statistically significant difference between the two subgroups (*p* > 0.05).

## Data Availability

The data used to support the findings of this study are available from the corresponding author upon request.
